# Internet-Delivered Dialectical Behavioral Therapy Skills Training for Suicidal and Heavy Episodic Drinkers: Protocol and Preliminary Results of a Randomized Controlled Trial

**DOI:** 10.2196/resprot.7767

**Published:** 2017-10-25

**Authors:** Chelsey Wilks, Qingqing Yin, Sin Yee Ang, Brandon Matsumiya, Anita Lungu, Marsha Linehan

**Affiliations:** ^1^ Behavioral Research and Therapy Clinics Department of Psychology University of Washington Seattle, WA United States; ^2^ Department of Psychology University of Central Florida Orlando, FL United States; ^3^ Lyra Health Burlingame, CA United States

**Keywords:** dialectical behavioral therapy, randomized controlled trial, eMental health, suicide, heavy episodic drinking, emotion dysregulation

## Abstract

**Background:**

The need to develop effective and accessible interventions for suicidal individuals engaging in heavy episodic drinking (HED) cannot be understated. While the link between alcohol use and suicidality is a complex one that remains to be elucidated, emotion dysregulation may play a key role in alcohol-related suicide risk in these individuals.

**Objective:**

In the current study, an 8-week Internet-delivered dialectical behavior therapy (DBT) skills training intervention was developed and preliminarily evaluated for suicidal individuals who engage in HED to regulate emotions. The aim of the study is to evaluate the feasibility and effectiveness of the therapist-assisted and Internet-delivered intervention, and to inform the design of a subsequent full-scale study.

**Methods:**

The study was a pilot randomized controlled trial comparing participants receiving immediate-treatment (n=30) to waitlist controls (n=29) over a period of 16 weeks. Intervention effects will be assessed longitudinally using hierarchical linear modeling and generalized estimating equations, along with analyses of effect sizes and clinically significant change. The primary outcomes are suicidal ideation, alcohol problems, and emotion dysregulation. Secondary outcomes include alcohol-related consequences, reasons for living, skills use, and depression.

**Results:**

The trial is ongoing. A total of 60 individuals returned their informed consent and were randomized, of whom 59 individuals were intended to treat. A total of 50 participants in the study were retained through the 16-week enrollment.

**Conclusions:**

There is a dearth of evidence-based treatment for individuals presenting with high risk and complex behaviors. Furthermore, computerized interventions may provide a beneficial alternative to traditional therapies. The particular clinical features and treatment needs of suicidal individuals who also engage in HED constitute key domains for further investigation that are needed to consolidate the design of appropriate interventions for this high-risk population.

**Trial Registration:**

Clinicaltrials.gov NCT02932241; https://clinicaltrials.gov/ct2/show/NCT02932241 (Archived by WebCite at http://www.webcitation.org/6uJHdQsC2)

## Introduction

Suicide is the tenth leading cause of death across all age groups and is among the top four causes of death for Americans aged 10 to 54 years [1]. Furthermore, approximately 40% of suicides were found to be preceded by acute alcohol use [2]. Alcohol intoxication, both acute and chronic, has been identified as a uniquely salient risk factor for suicide among individuals with suicidal ideation [3-6]. These findings bring attention to the importance of attending to problematic alcohol use, and binge drinking in particular, in the management of suicide risk.

One possible explanation for the prevalence of suicide attempts preceded by binge drinking (ie, heavy episodic drinking; HED) is that suicidal heavy drinkers lack emotion regulation capabilities [7-10]. Emotion dysregulation is associated with risk for both suicide and problematic drinking [11-13]. Furthermore, suicide risk may be particularly elevated in individuals who engage in HED to reduce acute negative affect [9]. Given the behavioral complexity of suicidal individuals who drink alcohol to regulate emotions, an alcohol-focused treatment may not be sufficient to increase coping skills and/or distress tolerance techniques. This problem suggests the need for novel treatment approaches for HED and suicide that focus on increasing emotion regulation capabilities.

Dialectical behavior therapy (DBT) [14-16] is a behavioral intervention with strong empirical evidence in reducing suicidal behavior [17]. DBT was specifically designed for high risk, complex individuals, and consists of a combination of individual psychotherapy, group skills training, telephone coaching, and a therapist consultation team. Recent evidence has found that the DBT skill-training component alone is efficacious at reducing dysfunctional behaviors associated with emotion dysregulation [18,19], and that DBT skills usage mediates reductions in a variety of outcomes (including suicidal acts) [20]. Taken together, a DBT skills training intervention is a potent and efficient treatment package.

Unfortunately, despite the existence of evidence-based interventions such as DBT, there remains a vast discrepancy between the need and availability of treatment for suicidal individuals with cooccurring addictive disorders. For example, the majority of those who engage in suicidal behaviors never engage in treatment [1,21]. In addition, a rare study on alcohol-dependent suicide attempters who were hospitalized following a suicide attempt found that only 6% of participants had been enrolled in psychotherapy prior to hospitalization, and only 9% went on to receive psychotherapy in the month after discharge [22]. Interestingly, while severity of suicidal ideation has been associated with low endorsement of formal treatment engagement, suicidal individuals may be more apt to utilize such nontraditional modalities, including online and Web-based interventions [23]. For these individuals, technological mediation may offer a convenient and viable means to access needed treatment.

Technologically-assisted interventions for psychological disorders constitute the focus of a growing body of research [24-26]. Internet-delivered or computerized interventions are available to anyone with an Internet connection, and can be accessed at relatively low costs. Moreover, attitudinal barriers (eg, stigma associated with psychiatric diagnoses and attending face-to-face therapy) can be circumvented by treatment delivered anonymously in one’s home. However, although computerized interventions have shown preliminary promise in accessing and treating suicidal ideation [27,28], as well as strong evidence in reducing problematic alcohol use [29-30], they are rarely tested on high risk individuals presenting with complex and multiple disorders [31].

To address this treatment gap, an Internet-delivered DBT skills intervention targeting the underlying mechanism of emotion dysregulation was adapted for individuals who endorse suicidal thoughts and engage in HED. The current pilot study is a proof of concept study that seeks to evaluate the feasibility, acceptability, and preliminary efficacy of a DBT skills training intervention delivered via technology (ie, Web-based portal). The following aims are: (1) to determine the feasibility of recruiting clients, administering the treatment, and retaining clients in the treatment; (2) to evaluate the safety of the treatment with respect to potential adverse events; (3) to assess the feasibility of the research methodology (eg, reliability of the measures used, feasibility of random assignment to treatment, appropriateness of the control condition); and (4) to evaluate the feasibility of using a wait-list control group in this line of research, and changes over time among individuals in such a comparison group.

## Methods

This study is a pilot randomized controlled trial (RCT) comparing an Internet-delivered Dialectical Behavioral Therapy Skills Training (iDBT-ST) to a waitlist control condition. Participants were randomized to begin the 8-week intervention either immediately or after an 8-week waiting period. Assessments included monthly batteries, weekly surveys, brief daily reports, and acceptability questionnaires, all of which were all self-assessed. The project’s primary aim is to determine whether further revisions of the iDBT-ST intervention are needed, and to inform the design of a subsequent full-scale RCT.

### Participants

Participants were recruited from ads placed online (eg, Craigslist, Reddit) and in a local newspaper containing a brief description of the study. Interested participants were phone screened for eligibility on alcohol use severity, frequency of binge drinking episodes, suicidal ideation, emotion dysregulation, mental health treatment history, and demographic characteristics. Inclusion criteria included: (1) being 18 years or older, (2) endorsing suicidal ideation in the past month (defined as endorsing a wish to die at least once in the past 30 days), (3) engaging in at least two episodes of heavy drinking in the past month, (4) high emotion dysregulation (defined as scoring one standard deviation above the normative mean on the 16-item Difficulties in Emotion Regulation Scale [32]), and (5) residing in the United States. Exclusion criteria included: (1) currently receiving psychotherapy; (2) diagnosis of bipolar I, schizophrenia, or schizoaffective disorder; (3) non-English speaking; (4) unable to read or write; and (5) having no access to a computer connected to the Internet. Of 398 individuals who expressed initial interest, 60 of 91 eligible individuals returned the informed consent forms and were randomized into the immediate-treatment (n=31) and waitlist (n=29) groups. One person randomized to the immediate-treatment group did not complete any of the assessments, leaving 59 people included in the analyses. The phone screening process helped us prevent participants from enrolling with multiple identities.

### Ethics and Consent

The study has been approved by the Human Subjects Division of the Institutional Review Board at the University of Washington (IRB# 50295). Through the initial phone screening, all interested individuals were briefed on the aims of the study and their rights as participants. Participants were informed that their answers to the questions were voluntary, and that they could withdraw from the study at any time. Informed consent forms (see Multimedia Appendix 1) containing details about the study, procedures, and possible risks and benefits involved were provided to all eligible participants. Prior to randomization, participants were required to indicate on the consent forms their preferred mode of communication for receiving study materials, and to return their signed consent forms to the study site. Referral resources for treatment and crisis services were sent by email to interested callers who were deemed ineligible for the study. All participants consented to have their nonidentifiable data published. Access to the dataset is restricted to the principal investigator and authorized research assistants only. Complete data from this study can be obtained by contacting the first author once data analysis has been completed. iDBT-ST is not currently available online.

This study focuses on sensitive topics such as alcohol use and suicidal behavior, and the phone screen and outcome assessments contain questions that could potentially be distressing to participants. As a safety measure, participants were encouraged to consult with research staff whenever they felt distressed during screening and later participation. Suicide risk was also monitored and addressed in ways that are discussed later in this paper.

### Procedure

Semistructured phone screening interviews were conducted with individuals who initiated contact via phone or email and remained interested after receiving information about the study. Phone screens were either prescheduled or conducted during the first call of contact, depending on caller preference, and were administered by trained research assistants. Eligible individuals received informed consent forms to sign and return, and were randomized once their signed forms were received at the study site.

Following randomization into the immediate-treatment and waitlist groups, participants in both conditions received a baseline assessment, followed by identical monthly assessments in the ensuing four months. Primary monthly outcomes included suicide ideation (Scale for Suicidal Ideation [33]), severity and frequency of alcohol consumption (Alcohol Use Disorders Identification Test; AUDIT [34], and Self-Administered Web-Based Timeline Followback [35,36]), and emotion dysregulation (Difficulties in Emotion Regulation Scale [37]). Secondary outcome measures included the Reasons for Living Inventory [38], Short Inventory of (Alcohol) Problems (SIP) [39], Patient Health Questionnaire [40], and Dialectical Behavior Therapy - Ways of Coping Checklist [41]. All participants also received brief online weekly assessments on suicide ideation and alcohol consumption. Suicide risk was monitored via weekly assessments.

Prior to treatment, participants also responded to their expectancy of outcome along with the credibility of the intervention (Credibility/Expectancies Questionnaire [42]). At the end of the treatment period, participants reported on their treatment satisfaction (Client Satisfaction Inventory-Short Form [43]) and therapeutic alliance (California Psychotherapeutic Alliance Scale [44]). Participants actively receiving treatment also received brief daily surveys about their urges to quit treatment, use of DBT skills, and engagement level.

All assessments were administered through an online survey platform (Qualtrics [45]) made accessible to participants via Uniform Resource Locator links in scripted emails sent at scheduled timepoints. As compensation for their participation, participants received up to US $120 in Amazon gift cards upon completing each monthly survey (baseline=$15, 4-week=$20, 8-week=$25, 12-week=$30, and 16-week=$30).

### Randomization

Eligible and consenting participants were randomized into the immediate-treatment (n=31) and waitlist groups (n=29). To control for potential covariates that may spuriously affect analytic results, an open source desktop application called MinimPy [46] was used to match participants for equal distribution between conditions on three variables. The three variables were: sex assigned at birth (Demographic Data Schedule), severity of suicide ideation (Suicide Behavior Questionnaire-Revised) and degree of disordered drinking (AUDIT).

### Managing Risk

Suicide risk was carefully monitored in the current study. Participants were assessed at the start and end of the recruitment phone screen, as well as throughout the course of their participation. High risk of suicide during phone-screening was indicated by endorsing 4 or more points on a 7-point Likert scale, or an increase of 3 or more points from the start of screening on urge to die by suicide. Throughout the 16-week trial period, weekly questionnaires assessing suicide risk were emailed to all participants. These emails included prompts on: (1) frequency of suicidal urges in the past week, (2) intensity of suicidal urges in the past week, (3) seriousness of acting out on suicidal urges in the past week, and (4) current suicidal urge, all using 5-point Likert scales. Participants endorsing a rating of 3 or higher on any item, or a 2-point increase from the previous week, would be called by phone and assessed for suicide risk. Additionally, the number for the National Suicide Prevention Lifeline was situated next to prompts on suicidality received by all participants.

The principal investigator was on call to intervene by phone at any time. Evidence-based procedures based on the University of Washington Risk Assessment Protocol (UWRAP) [47] were implemented by the principal investigator whenever further assessments of risk were warranted. The UWRAP guides the assessor through a suicide risk assessment procedure that evaluates the presence of a plan, access to means, likelihood of following through with a plan, being interrupted by others, et cetera. If a participant was determined to be at high risk based on this interview and the clinical judgment of the principal investigator, a safety plan was to be developed with the cooperation of the participant and the principal investigator. If a participant was unavailable by phone, a nonjudgmental *caring email* (see Multimedia Appendix 2) was sent, which expressed concern and encouraged participants to reach out to a suicide hotline or textline provided in the email.

### Intervention

One primary aim of iDBT-ST is to replace maladaptive behavioral strategies with alternative strategies for coping and emotion regulation. The current intervention comprises the skill-training component alone, which is administered in a computerized format. The intervention was modified from an online DBT skills training intervention for emotion dysregulation [48].

Participants randomized into the iDBT-ST group were immediately enrolled into the online treatment program, and received weekly sessions over a period of 8 weeks. After completing the eighth session, participants in active treatment were transitioned into a follow-up phase that entailed monthly and weekly assessments without treatment. Participants randomized into the waitlist group received repeated monthly assessments as well as weekly surveys for 8 weeks prior to receiving treatment.

**Figure 1:**
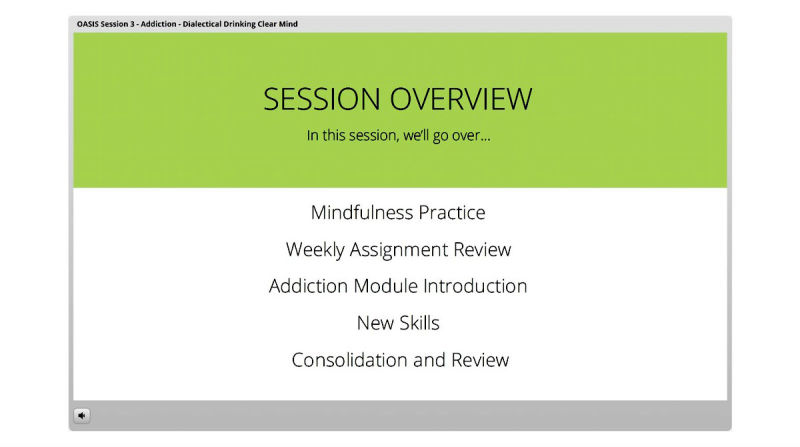
Screenshot of the session overview.

**Figure 2:**
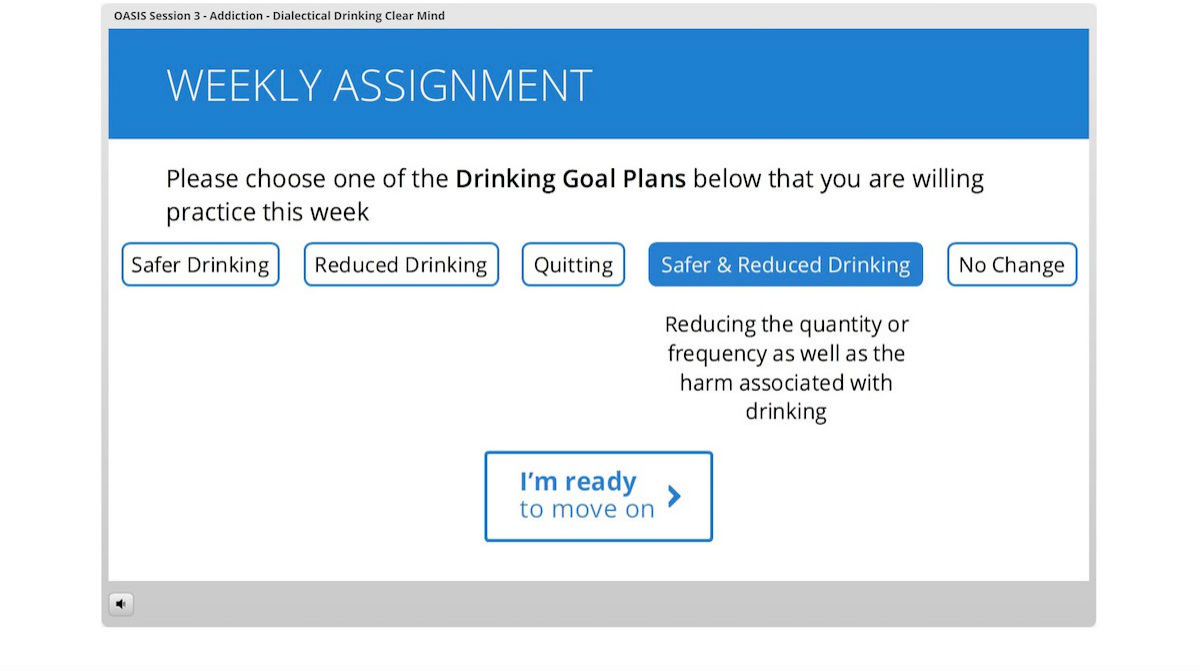
Screenshot of interactive activity (selecting drinking goal).

**Figure 3:**
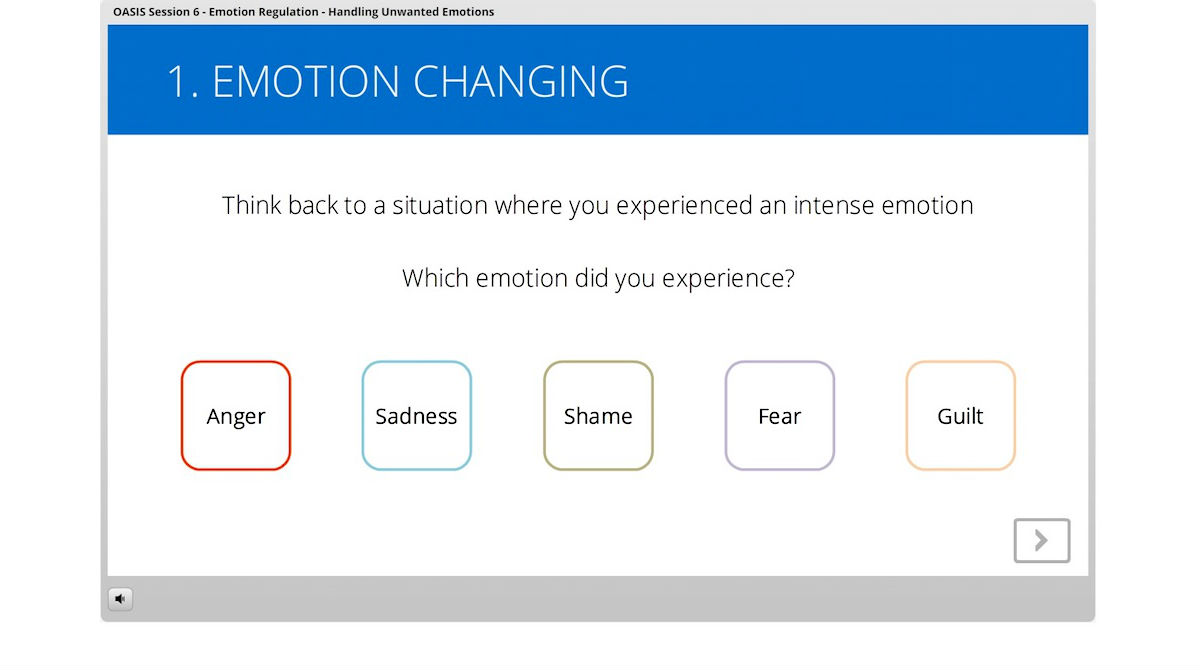
Screenshot of interactive activity (changing emotions).

The iDBT-ST intervention was administered through an online electronic learning server (Articulate Online [49]) which consists of 8 sessions (Table 1) accessible through an access portal individualized for each participant, with one new session activated each week. The sessions begin with a weekly assessment, followed by (1) a review of the last session at weeks 2-8, (2) an introduction of the current session, (3) skills teaching, and (4) summary and consolidation. The skills were taught via videos and graphics describing the skills and examples, along with interactive activities. The intervention completion rate of each participant was also monitored through the server. If consented by participants, reminders to complete sessions were sent via their preferred method before the next session became available. Individualized assignments of skill practice were provided following the completion of each session. See Figure 1, Figure 2, and Figure 3 for screenshots of the intervention.

**Table 1:** Description and function of session contents.

Session	DBT module	Skills	Function
1	Mindfulness	Observing, Describing, Participating	To introduce the foundational skills to develop nonjudgmental awareness of the present
2	Mindfulness	Nonjudgmentally, One-Mindfully, Effectively	To teach how to practice mindfulness with skillful effectiveness
3	Addiction	Dialectical Drinking, Clear Mind	To help learners find a middle path between oppressive sobriety and unrestrained freedom of drinking, and develop a clear mind
4	Addiction	Community Reinforcement, Burning Bridges	To teach strategies to identify relationships and activities that aim to stop or reduce problematic drinking
5	Emotions Regulation	Understanding Emotions	To teach the functions of emotions, and how to describe them
6	Emotions Regulation	Handling Unwanted Emotions	To teach skills to reduce frequency and quantity of unwanted emotions
7	Emotions Regulation	Building Mastery and Copy Ahead	To teach skills to build future resilience against intense emotion
8	Distress Tolerance	TIP (Temperature of face, intense exercise, paced breathing), Distract	To teach skills that help weather crises and intense negative emotions

**Figure 4:**
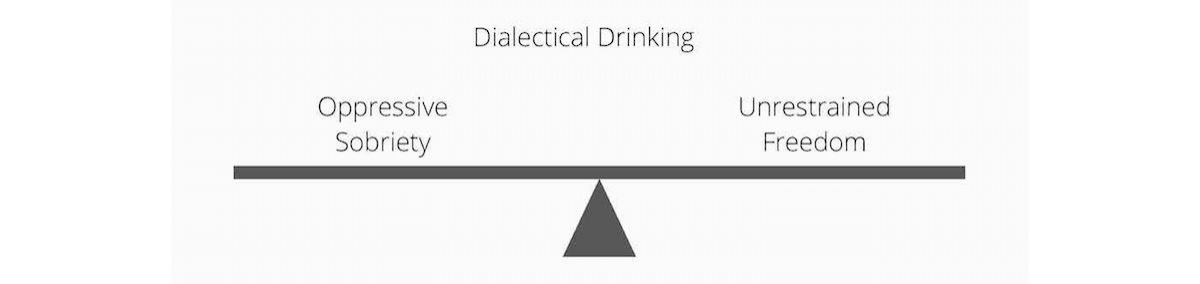
New Dialectical Behavioral Therapy skill: dialectical drinking.

Sessions 3 and 4 were designed to target problematic drinking, and included the new skill *Dialectical Drinking*. The skill of dialectical drinking describes the abstinence violation effect (see Figure 4), goal setting, pros and cons of maintaining or changing their drinking behavior, as well as an assessment of motivation and confidence to follow-through on their drinking goal. Dialectical drinking incorporates aspects of motivational interviewing [50] and harm reduction [51] as ways to nonjudgmentally reinforce a change toward goal-oriented drinking behavior. Problematic drinking is also targeted through the DBT skills of *Community Reinforcement*, whereby participants identify people, activities, and places that can reinforce their drinking goal, as well as *Burning Bridges*, whereby participants eliminate people, activities, and places that would reinforce their problematic drinking. In burning bridges, participants learned a variety of methods to manage alcohol cravings [15,16]. See Table 2 for common alcohol reduction strategies and their DBT counterparts.

**Table 2:** DBT skills that target problematic drinking.

Alcohol Reduction Strategy		Skill
**Deciding to make a change**		
	Goal setting	Dialectical Drinking
	Decisional balance	Dialectical Drinking
	Assessment of motivation and interest	Dialectical Drinking
	Abstinence violation effect	Dialectical Drinking and Clear Mind
	Tracking behavior	Daily diary card
**Making a change**		
	Contingency management	Contingency Management and Burning Bridges
	Craving management	Burning Bridges
	Drink refusal strategies	Dialectical Drinking

**Figure 5:**
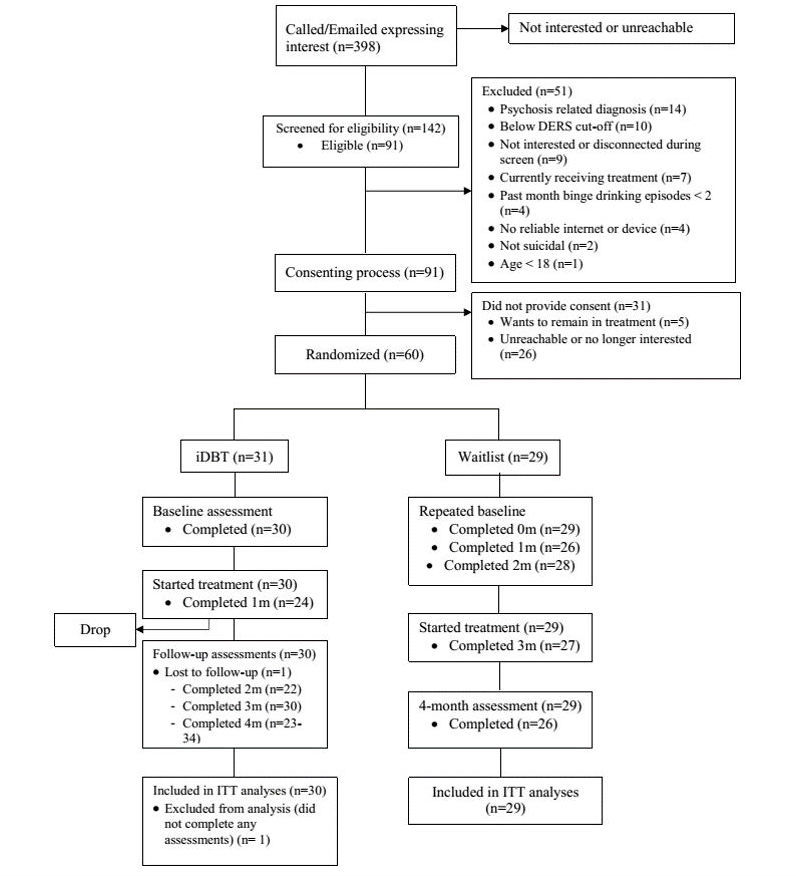
CONSORT diagram. DERS: Difficulties in Emotion Regulation Scale; iDBT: Internet-delivered Dialectical Behavioral Therapy; ITT: intention-to-treat.

### Confidentiality

All information provided by participants will be strictly confidential. Answers to phone screens and online assessments that will be kept indefinitely for research purposes were coded in a nonidentifying way, and stored in password-protected files on an encrypted server accessible only to the principle investigator and trained research assistants. Confidential records linking participant names with their respective research identification number will be deleted no later than September 30^th^, 2018. All identifying information from ineligible individuals was deleted immediately after phone screening. Additionally, the study site is a secure research clinic equipped with noise-cancelling devices to prevent conversations with participants from being overheard. Voice messages left for participants were devoid of details about the study and other sensitive information that may compromise confidentiality. Any paper documents that contained confidential Patient Health Information are kept in a locked area in the study site where access is restricted, and will be shredded before disposal. These documents may be examined in strictly confidential checks conducted by government or university staff for legal and safety insurance.

Participants were informed of one scenario in which confidentially may be breached. That is, if a participant reveals an imminent high risk of danger to their own or someone else’s life, research staff may address that risk to police or emergency resources.

### Power Calculation

A power analysis was not conducted because the primary purpose of the data analyses is to describe the effect of iDBT-ST protocol treatment over time using repeated observation data in a preliminary manner. The results will be used to help determine if further research on the treatment program for suicidal heavy drinkers is necessary in larger samples.

### Analyses

Intervention effects will be evaluated by examining change outcomes as a function of treatment condition using both hierarchical linear modeling (HLM) [52] for outcomes with a normal distribution and generalized estimating equations (GEEs) [53,54] for count outcomes. Both HLM and GEEs accommodate observations that are missing at random, thus increasing the power of analyses. Analyses will be conducted using an intent-to-treat framework, in which all randomized and available participants will be included in the analyses. To examine changes over time for the entire sample, analyses will be conducted among all participants during all assessment waves (baseline to 16-week). A set of analyses will also be conducted using the first three assessment waves (baseline to 8-week) comparing immediate-treatment to no treatment, and at each condition’s relative pretreatment, midtreatment (4-week for iDBT-ST; 12-week for waitlist), and posttreatment (8-week for iDBT-ST; 16-week for waitlist) assessment phases.

Given the pilot nature of the study, all outcomes will be evaluated in terms of effect sizes as well as clinically significant change using Jacobson and Truax’s specifications [55]. Treatment effect sizes will be analyzed using Cohen’s *d* and Relative risk ratios for continuous and count outcomes, respectively. Cohen’s *d* will calculate within-condition effect sizes across all assessment waves. Descriptive analyses will also be conducted to examine acceptability.

## Results

Recruitment to the study began June 17, 2016 and ended September 14, 2016. Data were collected using direct entry of online surveys tools (Qualtrics [45], Articulate Online [49]) by February 2017, and are currently undergoing analyses. The CONSORT diagram (Figure 5) presents the flow of participants during recruitment, enrollment, and participation. Of the 398 individuals who expressed interest in participating, 91 were determined to be eligible through the screening process. A total of 60 individuals returned their informed consent and were randomized, of whom 59 individuals were intended-to-treat. A total of 50 participants in the study were retained through the 16-week enrollment.

## Discussion

The primary aim of this trial is to preliminarily evaluate an Internet-delivered DBT skills training intervention for suicidal and heavy episodic drinkers. Research on suicidal individuals who engage in HED is paramount to understanding and managing suicide risk, particularly as it pertains to high-risk populations who are less inclined to seek formal treatment. The set of protocols used in DBT to manage acute and chronic suicidality was translated relatively seamlessly for use in this pilot study’s Web-based approach, demonstrating their potential utility as tools for remote risk-management in computerized interventions. A concern of this treatment is the 8-week waiting period; however, waiting times are often a reality for individuals seeking assessment and treatment (eg, mean=65.4 days) [56] due to resource or staff shortages [57-60], and we believe we can answer an important question about placing high risk individuals on waiting lists. Participants were fully informed about the possibility of being randomized to an 8-week waiting list, as well as their rights to withdraw from the study at any time. Referrals to treatment and crisis services were provided whenever participation was declined or withdrawn.

One major aim of the study was to investigate the feasibility of recruiting and retaining participants from this population. Sixty eligible individuals were recruited within three months, demonstrating the feasibility and effectiveness of the online recruitment strategy. Retention rates, particularly for waitlisted participants, were anticipated as a potential challenge, with findings in the literature suggesting an association between longer wait periods and reduced treatment motivation and retention [61,62]. To address this problem, customized reminders were sent to all participants to encourage treatment engagement and assessment completion [63]. Finally, 83.3% (50/60) of all participants were retained in the study by the end of their enrollment, suggesting that customized reminders may be an effective strategy to mitigate study dropout rates.

The level of risk commonly attributed to such studies may be inhibitive to research. Nevertheless, grounds for the safeguarding of participant safety can be established with careful implementation of validated risk-management protocols, which equip researchers with contingencies against anticipated harm in conducting much-needed studies. In addition, the feasibility of recruiting, retaining, and monitoring participants demonstrated in this pilot study provides preliminary suggestions for the developing field of research on technologically-assisted interventions that may become beneficial alternatives to traditional therapy, or mediate adaptive help-seeking in at-risk and underserved populations.

## Appendix

Multimedia Appendix 1 Informed consent.

Multimedia Appendix 2 Caring email sent to those unreachable by phone.

## Abbreviations

AUDIT: Alcohol Use Disorders Identification Test
DBT: dialectical behavior therapy
GEE: generalized estimating equation
HED: heavy episodic drinking
HLM: hierarchical linear modeling
iDBT-ST: Internet-delivered Dialectical Behavioral Therapy Skills Training
NIAAA: National Institute on Alcohol Abuse and Alcoholism
RCT: randomized controlled trial
UWRAP: University of Washington Risk Assessment Protocol
