# Supplementary material for: Internet-Delivered Dialectical Behavioral Therapy Skills Training for Suicidal and Heavy Episodic Drinkers: Protocol and Preliminary Results of a Randomized Controlled Trial
Source: JMIR Res Protoc. 2017 Oct 25;6(10):e207. doi: 10.2196/resprot.7767 (PMC5677770; doi:10.2196/resprot.7767)
Supplement: Multimedia Appendix 1 [file resprot_v6i10e207_app1.pdf]

Appendix 1. Informed Consent

University of Washington
Behavioral Research and Therapy Clinics
Department of Psychology, Box 351525
Seattle, WA 98195-1525
(206) 543-BRTC

ASSESSMENT AND TREATMENT CONSENT FORM

iDBT-OASIS Treatment Study

Principal Investigator & 24-hour Emergency Contact
Chelsey Wilks, M.S., Graduate Student
Department of Psychology, BRTC (206) 616-1508
24-hour Emergency Contact (206) 314-6394

INVESTIGATOR'S STATEMENT

We are asking you to be in a research study. The consent form gives you the information you need to decide whether to be in the study. Please read the form carefully. You may ask us any questions about the purpose of the research, what we would ask you to do, the possible risks and benefits, your rights as a volunteer, and anything else about the research or this form that is not clear. When all your questions have been answered, you can decide if you want to be in the study or not. This process is called 'informed consent'. We will give you a copy of this form for your records.

PURPOSE OF THE STUDY

We want to evaluate a specific type of psychotherapy for persons who have thoughts of ending their life, engage in heavy episodic drinking, and have difficulty regulating emotions. The therapy, Dialectical Behavior Therapy (DBT) skills training, will be delivered by computer. Face-to-face DBT helps people with these problems. However, it has not been evaluated in a computerized format for suicidal heavy drinkers.

STUDY PROCEDURES

Overview

If you agree to be in the study, we will assign you randomly (by chance) to either receive the therapy immediately, or wait for eight weeks and then receive it. Study participation is 16 weeks. We are asking you to commit to your assigned treatment for 8 weeks either immediately or after an 8 week waiting period. Study participation also includes five monthly assessments and 16 weekly questionnaires. Also, we ask that you complete daily reports for the 8 weeks while you are receiving therapy. Below is more detail.

Assessments

All assessments will be completed online.

- **Monthly assessments** (45-60 minutes) concerning problems with controlling emotions, about how you experience certain feelings, ways you handle problems, drug and alcohol use, and what you expect and think about treatment.
- **Weekly questionnaires** (5 minutes) asking you about urges to die and patterns of alcohol use.

- **Daily reports** (2-3 minutes) rating skills practice (only during active treatment).

Treatment Description
You will be randomly assigned (by chance) to receive the treatment either right away or after an 8-week waiting period. You will receive Dialectical Behavior Therapy Skills Training by computer at no cost lasting 8 weeks, approximately one 45 to 60-minute session per week. We will also send you daily mail or text messages to encourage you to practice the DBT skills you learn through the online program.

You will not receive medication as part of this study, but if you are already on psychotropic medication, we encourage you to continue seeing your regular prescriber for ongoing care. If you are already on medication we are asking you to stay on the same dose throughout the treatment portion of the study, and if you are not taking any psychotropic medication to not start it throughout the study. You should talk with your prescriber about whether this is appropriate for you.

If you are currently seeing another counselor, case manager, or psychotherapist, we are asking you to stop seeing them for the duration of the study. You may resume seeing them after the last follow-up assessment is completed. We also ask you not to start seeing a new counselor, case manager or psychotherapist while you're still in treatment with us.

If you are in the waitlist group, you will wait 8 weeks and then will receive the therapy described above. You will also complete the five monthly assessments as well as 16 weekly questionnaires.

Electronic communication as part of treatment
As part of this project we ask you to consider receiving prompts and reminders from us in order to support you in practicing in your daily life what you learn in our online intervention.
We will send you 2-3 messages every day:

- One prompt for practicing a specific skill or assignment
- One message for reminding you to track your skills practice through a brief online survey (the link for the online survey will be included in the message)
- Sometimes a reminder to login to the online intervention if it has been a while since your last login or if a new session is available.

We think receiving these prompts and reminders will help you benefit more from the therapy. You can choose to not receive these messages although we advise you to at least try them. You can e-mail us to change your mind about receiving the messages and about how you receive them.

In summary, we ask you to agree to the following during the study:

1. Commitment to be in the study for 16 weeks which includes 8 weeks of treatment;
2. If you are on psychotropic medication, staying on the same medication & dose. If you are not on medication, not start it during the study. You might want to consult a provider about whether this decision is appropriate for you;
3. If you are seeing another counselor or therapist, not see them and not seek additional professional mental health treatment during the study unless approved by our research team;
4. Complete all 5 monthly assessments during the 16 week study;
5. Complete five-minute weekly questionnaires that will include ratings on your suicidal urges and drinking patterns;
6. Complete a daily 2-3 minute report of the skills you practice while in therapy;
7. Receive daily prompts via your preferred method of communication (email or text message (SMS) and check your preferred method of communication regularly to receive the prompts.

GENERAL RISK ASSOCIATED WITH SCREENING, ASSESSMENTS, AND TREATMENT

The assessment and questionnaires may be stressful or emotionally uncomfortable for some people, but others find this a useful way to express some of their feelings and gain helpful information about them. Other risks may include fatigue from the lengthy assessments. We try to minimize this by stopping whenever you want to and by having breaks. Please note that your responses to any of the assessments will not be seen immediately by study staff. Therefore, we will provide a number to a national crisis hotline following question on suicidality if you need immediate assistance. During each of the monthly assessments we encourage you to call us at the study number (206) 616-1508 in case you are experiencing any distress from answering the questions. We do our best to get back to you by the end of the business day or 24 hours; however, if you require immediate help for a life threatening emergency we encourage you to call 911.

Breach of confidentiality is a risk to being in the study if your information was accidentally given to or was taken by someone who should not have it. For example, someone could overhear you talking to our team or hear a phone message. Another example is if someone else reads a letter from our clinic intended for you. We try to prevent these breaches of confidentiality by making sure that assessors and therapists only leave their names, the name of the clinic ('Behavioral Research and Therapy Clinics') and the name of the institution ('University of Washington') voicemail messages and that they use general University of Washington envelopes.

You were selected for this study because you have suicidal thoughts, drink alcohol to cope, and have problems with regulating emotions. The treatment study is designed to help you with these problems. Our approach is to provide you with knowledge and support to make your life into what you want it to be. Making needed changes and confronting the painful aspects of yourself, your past, and your current life may be necessary in order to achieve this. However, this can be extremely difficult and stressful. As a part of this treatment program, you are also asked to discontinue treatment with all non-study mental health treatment providers. Reducing or discontinuing contacts with other mental health professionals may also cause increased emotional discomfort, especially at first.

Using Email/SMS (text) messages has some risks including but not limited to:

- Messages may be forwarded, printed, and stored in numerous paper and electronic forms.
- Messages may be sent to the wrong address by any sender or receiver.
- Messages may be easier to forge than handwritten or signed papers.
- Copies of messages may exist even after the sender or the receiver has deleted his or her copy.
- Service providers have a right to archive and inspect messages.
- Messages may be intercepted, altered, or used without detection or authorization.
- Messages may spread computer viruses.
- Message delivery is not guaranteed.

The use of Email/SMS (text) messages in this study is intended as a one way communication from the study to you in order to provide you with prompts or reminders. It is not intended in this study to receive information from you and for this reason you should not respond to our messages. Should you respond to any of these messages your answers will NOT be monitored by the research team. Because of this it is important that you DO NOT use Email/SMS (text) messages to communicate to us in an emergency or to send time-sensitive information to us.

PERMISSION TO FIND YOU

If we are unable to reach you in the future, we would like your permission to try to locate you during the study. We will use public info such as yellow and white pages, and would like your permission to use other ways to locate you.

SCHEDULE OF AND PAYMENT FOR ASSESSMENTS

You will be paid $15-30 (in Amazon.com gift cards) to complete each of the 5 monthly assessments. No additional payment is provided for completing the weekly questionnaires or daily reports.

Assessment	Amount
1st week	$15 (in Amazon.com gift card)
4th week	$20 (in Amazon.com gift card)
8th week	$25 (in Amazon.com gift card)
12th week	$30 (in Amazon.com gift card)
16th week	$30 (in Amazon.com gift card)

ALTERNATIVES TO TAKING PART IN THIS STUDY

If you are not eligible for the study or decide not to take part, there may be other treatments available in the community. We will give you a list of referrals to other mental health services/providers, including low cost and sliding scale providers. As an alternative to this study, you may choose to pursue these other treatment options. This study will not pay for these.

BENEFITS OF THE STUDY

You may benefit from being in this study because you will be receiving 8 weeks of no-cost computerized DBT skills training treatment to increase emotion regulation, reduce problematic drinking, and reduce suicidal thoughts. The findings of the study may contribute to our understanding and treatment of people with these difficulties and may help other people with similar types of problems in the future.

SOURCES OF FUNDING FOR THIS STUDY

Funds for this study have been provided from the University of Washington Alcohol and Drug Abuse Institute and the National Institute on Alcohol Abuse and Alcoholism.

CONFLICT OF INTEREST

Dr. Linehan is the developer of the Dialectical Behavior Therapy (DBT) skills training being studied and may benefit financially, depending on the results of the study. This financial interest and the design of the study have been reviewed and approved by the University of Washington. A Conflict Management Plan was developed to minimize any possible effect of this financial interest on your safety or welfare. The Plan will also protect the quality and reliability of the research.

CONFIDENTIALITY

All information you provide us will be strictly confidential. Information you give us will be shared between research and clinical staff members of the BRTC on a 'need to know' basis. If information is to be exchanged with other mental health professionals outside the BRTC for case management, training or research purposes, you will be asked to sign a separate release of information form. If you refuse, information will not be released. However, if we have a strong reason to believe that you are in danger of

suicide or harming others, we will take steps to save your life or to intervene to protect others. We will keep identifying information in a secured but accessible location. In an emergency, this information will be accessible to clinical and research staff.

Any paper documents will be kept in a locked area of the research clinic where access is restricted to authorized BRTC research personnel only and will be stored in a separate locked area. Identifiable information about you will be destroyed no later than 9/30/2019. We will keep the de-identified data indefinitely.

Government or university staff sometimes review studies such as this one to make sure they are being done safely and legally. If a review of this study takes place, your records may be examined. The reviewers will protect your privacy. The study records will not be used to put you at legal risk of harm.

OTHER IMPORTANT INFORMATION

If at some point you were to drop out of treatment, monthly assessments will continue unless you tell us you do not want to be contacted again. If you drop out of assessment, you will be discontinued from treatment. More precisely you will not have access to intervention weeks 6, 7, 8 if you miss your mid-treatment 4 week assessment. If you choose to drop out of the study, we will provide you with referral information for providers of therapy and/or medication management. The study will not pay for this.

Some mobile phone providers charge an additional fee for the sending and receiving of SMS/text messages, so if you choose to receive messages below via SMS you might be charged additionally by your mobile phone provider. You can opt to only receive messages from us via e-mail or to not receive messages at all (although we think you will benefit from our intervention more via these messages).

Taking part in this research study is voluntary. Please take time to decide whether you want to be in it. You can refuse to take part, or can stop and withdraw at any time for any reason, without penalty or loss of benefits to which you are otherwise entitled. You are free not to answer any question or item that you do not wish to answer and are welcome to take breaks during the assessments and questionnaires. Should you choose to not continue in the study or any portion of it, contact the researcher at 206-616-1508.

RESEARCH RELATED HARM

It is important that you promptly tell the researcher if you believe that you have been harmed by this study. You can call the researcher at the number listed at the top of this form. For a life-threatening problem, call 911 right away or seek help immediately.

If you are harmed by the study and need treatment for this, the costs of the treatment may be billed to you or your health insurance just like other medical costs or it may be covered by the UW's discretionary Human Subjects Assistance Program (HSAP), depending on a number of factors. If you wish to request HSAP coverage yourself, contact the researcher or the UW Human Subjects Division at hsdinfo@uw.edu or 206-543-0098. Ask the researcher if you would like information about the limits and conditions of HSAP. The UW foes not normally provide any other form of compensation for injury. However, the law may allow you to seek payments for injury related expenses if they are caused by malpractice or the fault of the researchers. You do not waive any right to seek payment by signing this consent form.

SUBJECT'S STATEMENT

This study has been explained to me. I volunteer to take part in this research. I have had a chance to ask questions. If I have questions later about the research or feel that I have been harmed by it, I can ask the researcher listed above. She can be reached at 206-314-6394 or 206-616-1508. At any time, I may designate individuals or agencies that I do not wish the investigators to contact. If I have questions about my rights as a research subject, I can call the Human Subjects Division at (206) 543-0098. I will receive a copy of this consent form.

PLEASE INITIAL >**_______ I agree to not use Email/SMS messages for communicating with the study providers in an emergency or for sending time-sensitive information.**

_____ YES Please mark whether you would like to receive **EMAIL** prompts/reminders to practice skills and
_____ NO complete homework during the treatment portion of the study

_____ YES Please mark whether you would like to receive **TEXT** prompts/reminders to practice skills and
_____ NO complete homework during the treatment portion of the study

_____ YES Please mark whether we have permission to search for you if we are unable to locate you by _____ **NO** calling friends, relatives and other contacts you have given us to see if they know where you are. The people we call will be under no obligation to give us any information

_____ YES Please mark whether we have permission to search for you online on social networks such as _____ **NO** Facebook, LinkedIn, MySpace, and Twitter as well as any contact information we find on such websites to contact you. We will not post any information on such websites about you.

Subject's Printed Name: ____

Subject's Signature: ____

Date: ____
