# Supplementary material for: Internet-Delivered Dialectical Behavioral Therapy Skills Training for Suicidal and Heavy Episodic Drinkers: Protocol and Preliminary Results of a Randomized Controlled Trial
Source: JMIR Res Protoc. 2017 Oct 25;6(10):e207. doi: 10.2196/resprot.7767 (PMC5677770; doi:10.2196/resprot.7767)
Supplement: Multimedia Appendix 2 [file resprot_v6i10e207_app2.pdf]

Appendix 2. Caring email sent to those unreachable by phone

Dear _____ ,

Hope you're doing ok! Below are some resources if you need anyone to talk to, are in a crisis, or if you would like to reach out.

National Suicide Prevention Lifeline: 1-800-273-8255

Website with chat option:
http://www.suicidepreventionlifeline.org/

Crisis numbers by County and State:
http://www.suicide.org/suicide-hotlines.html
http://www.sprc.org/states

Crisis text line
http://www.crisistextline.org/

Best,
Chelsey
The OASIS Team
